# Implications of metabolism-driven myeloid dysfunctions in cancer therapy

**DOI:** 10.1038/s41423-020-00556-w

**Published:** 2020-10-19

**Authors:** Laura Strauss, Valentina Guarneri, Alessandra Gennari, Antonio Sica

**Affiliations:** 1 Department of Immunopharmacology and Immuno Oncology, EMD Serono Research & Development Institute, Inc., 801195 45A Middlesex Turnpike, Billerica, MA 01821 USA; 2 Department of Surgery, Oncology and Gastroenterology, University of Padova, Padova, Italy; 3 Veneto Institute of Oncology-IRCCS, Padua, Italy, Padova, Italy; 4 Division of Oncology, Department of Translational Medicine, University of Eastern Piedmont, Novara, Italy; 5 Department of Pharmaceutical Sciences, University of Eastern Piedmont, A. Avogadro, via Bovio 6, Novara, Italy; 6 Humanitas Clinical and Research Center, IRCCS, Via Manzoni 56, Rozzano, Milan Italy

**Keywords:** Myelopoiesis, Tumor-associated macrophages, Myeloid-derived suppressor cells, Metabolism, Cancer therapy, Immunosuppression, Cancer metabolism, Cancer microenvironment

## Abstract

Immune homeostasis is maintained by an adequate balance of myeloid and lymphoid responses. In chronic inflammatory states, including cancer, this balance is lost due to dramatic expansion of myeloid progenitors that fail to mature to functional inflammatory neutrophils, macrophages, and dendritic cells (DCs), thus giving rise to a decline in the antitumor effector lymphoid response. Cancer-related inflammation orchestrates the production of hematopoietic growth factors and cytokines that perpetuate recruitment and activation of myeloid precursors, resulting in unresolved and chronic inflammation. This pathologic inflammation creates profound alterations in the intrinsic cellular metabolism of the myeloid progenitor pool, which is amplified by competition for essential nutrients and by hypoxia-induced metabolic rewiring at the tumor site. Therefore, persistent myelopoiesis and metabolic dysfunctions contribute to the development of cancer, as well as to the severity of a broad range of diseases, including metabolic syndrome and autoimmune and infectious diseases. The aims of this review are to (1) define the metabolic networks implicated in aberrant myelopoiesis observed in cancer patients, (2) discuss the mechanisms underlying these clinical manifestations and the impact of metabolic perturbations on clinical outcomes, and (3) explore new biomarkers and therapeutic strategies to restore immunometabolism and differentiation of myeloid cells towards an effector phenotype to increase host antitumor immunity. We propose that the profound metabolic alterations and associated transcriptional changes triggered by chronic and overactivated immune responses in myeloid cells represent critical factors influencing the balance between therapeutic efficacy and immune-related adverse effects (irAEs) for current therapeutic strategies, including immune checkpoint inhibitor (ICI) therapy.

## Introduction

Enhanced myelopoiesis is recognized as the primary factor that drives inflammatory disorders, including cancer, and is characterized by aberrant differentiation of myeloid progenitors, with an accumulation of dysfunctional myeloid cells endowed with suppressive functions, including myeloid-derived suppressor cells (MDSCs), tolerogenic dendritic cells (DCs), and tumor-associated macrophages (TAMs).^1^

Hematopoietic stem cells (HSCs) activation in persistent low-grade inflammation in cancer or overactivation (i.e., in acute infections or sepsis) perpetuates and increases myelopoiesis at the expense of lymphopoiesis, leading to expansion of a pool of immature and dysfunctional myeloid cells^1^ that besiege and exhaust antitumor immunity, thus resulting in local and systemic host immunosuppression.^2,3^ This pathologic myelopoiesis, leading to pro-disease phenotypes, provides us with an unresolved immunological paradox to date, since enhanced myeloid recruitment and function in tumors or infections should represent the front line of host defense and avoid disease progression.

Multiple inflammatory insults drive “pathological myelopoiesis”,^4^ including pathogen-associated molecular patterns and damage-associated molecular patterns,^5^ which are sensed by pattern-recognition receptors.^6^ Innate immune cells activated through PPRs provide the source for cytokines and myelopoietic growth factors acting on myeloid progenitors. Among these cytokines, the pleiotropic cytokines IL-1, tumor necrosis factor (TNF), and interleukin-6 (IL-6) serve as key promoters of emergency myelopoiesis by controlling the dynamics of transcription factors involved in myeloid lineage fate decisions and function.^7^ Growing evidence suggests that key transcription factors of emergency myelopoiesis, such as PU.1, interferon regulatory factors, CEBP/beta and RORC, in addition to driving myelopoiesis, are expressed in adipose tissue and have a central role in adipocyte differentiation, adipose inflammation, and insulin resistance (IR).^8–10^ This sharing of transcription networks between the adipose tissue and myeloid cells indicates that alterations in metabolic homeostasis may have a profound impact on myelopoiesis and therefore coordinate immune responses to environmental cues. Interestingly, studies show that low-grade inflammation in the adipose tissue and liver of elderly individuals or patients with metabolic dysfunction triggers transcriptional networks that reprogram steady-state hematopoiesis towards persistent and myeloid-biased hematopoiesis.^7,11^ Therapeutic targeting of PU.1 on adipocytes and adipose and liver macrophages improves glucose homeostasis and reduces liver steatosis, inflammation, and fibrosis in mouse models of steatohepatitis,^12^ indicating that targeting regulators of emergency myelopoiesis in patients with metabolic inflammation may revert pathologic inflammation and restore tissue homeostasis. Evidencing a critical contribution of dysregulated transcriptional networks of myelopoiesis and immunometabolism to the outcome of immunotherapy, recent studies have shown that hyperglycemia and hypercholesterolemia induce long-lasting changes in the transcriptional landscape of HSCs and myeloid progenitors (MPs), which perturb myeloid lineage fate decisions and the functional polarization of myeloid cells,^13,14^ and these changes persist even after changing to a control diet and upon weight loss^15,16^. Studies support this novel concept by showing that resistance to cancer immunotherapy correlates with host intrinsic metabolic dysfunctions such as hormone imbalance, IR, changes in glucose and lipid metabolism and enhanced inflammatory mediators.^17^

Extensive research published in medical and scientific journals has demonstrated that cancer cell-intrinsic metabolism hijacks the regulation of antitumor immune signaling, contributing to immunotherapy resistance.^18,19^ However, the role of aberrant immune cell-intrinsic metabolism in immunotherapy resistance remains poorly investigated to date.

As the majority of patients still show de novo or adaptive resistance to current immunotherapies, identifying immunometabolic checkpoints that coordinate myelopoiesis and effector lymphoid responses to oscillations in metabolites and inflammatory signals has become a hot research topic for improving cancer immunotherapy and preventing immune-related adverse events (iRAES). In accordance with this scenario, increasing evidence shows dysregulated cellular signaling and metabolism in myeloid cell subsets that infiltrate immunologically cold tumors resistant to immune checkpoint inhibitors (ICIs), chemotherapy (CT), and radiotherapy, with the infiltrates characterized by a lack of T and NK cell infiltrates, and accumulation of MDSCs, TAMs, and tolerogenic DC.^3,20^

This review aims to highlight potential targets for myeloid therapy, with a specific focus on recent efforts combining myeloid-targeted immune therapy with strategies to restore metabolic homeostasis.

## Interconnection between metabolic syndrome and inflammation

Metabolic syndrome is a collection of disturbances, including glucose intolerance, obesity, hypertension, and dyslipidemia,^21^ with increasing prevalence in developed countries. Chronic low-grade inflammatory conditions have been implicated as a major factor for metabolic syndrome,^22^ which is accompanied by metabolically triggered inflammation,^23^ a condition that does not completely fit into the classical definition of acute or chronic inflammation. Systemic metabolic inflammation accelerates immune overactivation and dysregulation and can support profound immune deficiency. This dual role of metabolic inflammation suggests that the plasticity of immune responses is intricately linked to the intracellular metabolism of immune cells and is highly sensitive to systemic and local metabolic oscillations in tissues.

Recent findings have highlighted the substantial impact that metabolic syndrome has on lymphoid tissue integrity, lymphocyte development, phenotypes and activity, and the coordination of innate and adaptive immune responses. Importantly, these changes are associated with an overall negative impact on relapse-free survival in cancer patients.^24,25^ How exogenous and intrinsic metabolic signals affect the immune response in patients is poorly understood to date. Extensive clinical data and experimental models demonstrate the involvement of obesity and adipokines in the pathogenesis and treatment response of a broad variety of autoimmune diseases.^26^ Hyper and persistent secretion of inflammatory cytokines can cause IR in the adipose tissue, skeletal muscle and liver by inhibiting insulin signal transduction. Myeloid cells, primarily resident in colon, liver, muscle, and adipose tissue, serve as a source of chronic inflammation, termed meta-inflammation, causing localized IR via autocrine/paracrine cytokine signaling and systemic IR via endocrine cytokine signaling.^27^ In addition, meta-inflammation predisposes patients to overactivated immune responses, with co-occurring immune exhaustion and immunosuppression.^28^

Finally, the level of inflammation has a direct implication in therapy, affecting the plasticity of the immune system. Indeed, cancer therapy-induced inflammation (i.e., inflammation induced by CT and radiotherapy) is considered an additional mechanism reinforcing aberrant myelopoiesis. In this respect, IL-6 was shown to activate emergency myelopoiesis after myeloablation consequent to cytotoxic treatments.^29,30^ Furthermore, the use of monoclonal antibodies (i.e., nivolumab) targeting checkpoint inhibitors (i.e., PD-1) in cancer immunotherapy is frequently associated with severe side effects, which are mitigated by anti-inflammatory treatments, including steroids or the anti-IL-6 antibody tocilizumab in steroid-refractory patients.^31^ Combination therapy with nivolumab + ipilimumab was recently approved for treating unresectable cases of malignant melanoma. Despite the high response rate, this therapy is associated with a high incidence of serious adverse events, including immune-related hemophagocytic syndrome/hemophagocytic lymphohistiocytosis (irHPS/HLH), macrophage activation syndrome, and secondary HLH, a cytokine storm syndrome associated with multiorgan system dysfunction and high mortality rates.^32,33^ Therefore, understanding the role of inflammatory mediators and their interconnection with patient metabolic status will be necessary to ensure proper immunological manipulation and the best personalized therapies.

### The controversial role of metabolic syndrome-associated myeloid dysfunction in cancer

In response to immunologic stresses, including infection and cancer, hematopoietic stem and progenitor cells in the bone marrow (BM) sense peripheral inflammation and adapt through increased proliferation and skewing towards the myeloid lineage. Although these adaptations meet the need for more innate immune cells, this lymphoid-myeloid switch and the enhanced myelopoiesis might also perpetuate inflammatory and metabolic disorders by generating a feed-forward loop between inflammation-triggered MP cells and the inflamed tissue.^4^ Alterations in MPs, as well as the expansion of pro-inflammatory monocytes and MDSCs, also arise in high-fat diet (HFD)-induced obesity^34–36^ and are considered a biomarker for the risk of obesity-associated diseases, such as diabetes and atherosclerosis.^37,38^ Notably, it has been hypothesized that one of the mechanisms by which obesity promotes cancer mortality is through the induction of MDSCs.^39–41^ In accordance with this hypothesis, obese mice with renal cancer develop a robust immunosuppressive environment that is characterized by heightened local and systemic CCR2^+^ MDSC prevalence.^42^ In a pancreatic cancer mouse model of diet-induced obesity, cells expressing common neutrophil and MDSC markers (Gr1^+^CD11b^+^) were recruited to the pancreas by adipocytes and pancreatic stellate cells producing the pro-inflammatory mediator IL-1β. Depletion of Gr1^+^CD11b^+^ cells, IL-1β, or pancreatic stellate cells prevented the rapid growth of cancer in obesity.^43^ Similarly, in BALB/c mice carrying 4T1 mammary carcinoma, MDSCs from HFD mice were more immunosuppressive than MDSCs from low-fat diet (LFD) mice, correlating with higher tumor progression and reduced survival, and depletion of MDSCs in HFD mice restored activation of tumor-reactive CD8^+^ T cells, reducing tumor progression, and spontaneous metastasis to levels comparable to those seen in LFD mice.^44^ In addition to dampening the host-specific antitumor T cell and DC immune responses, metabolic syndrome-associated myeloid cells can drive pathologic overactivation of the immune response. The increased risk of developing intra-abdominal obesity and metabolic syndrome in the elderly population^45^ is associated with hyperactivated macrophages, persistent myelopoiesis, and MDSC expansion, which paradoxically can drive a deadly cytokine storm.^46^ This increase in inflammatory sensitivity leads to a mixed immunophenotype of hyperactivated and exhausted and tolerogenic immune cells that may predispose individuals to infection or CAR-T cell cancer therapy-induced cytokine storm. The role of MDSCs in immune dysregulation in patients with metabolic syndrome remains controversial, as MDSCs can also have beneficial effects and protect mice against metabolic dysfunction and inflammation.^47,48^ Nevertheless, metabolic syndrome-associated myeloid dysfunction, including the expansion of MDSCs, has been associated with persistent and unresolved myelopoiesis, leading to immune-mediated tissue pathologies such as fibrosis, tumor angiogenesis, and metastases.^49^ Therefore, therapeutic interventions that reprogram the metabolism of MPs and myeloid cells in tumor patients towards lineage fate decisions supporting myeloid effector and antigen-presenting functions may restore immune-mediated tissue homeostasis and decrease the risk of disease relapse.

### Metabolic cues can inform pathogenic myelopoiesis

Mounting evidence demonstrates a role for hypercholesterolemia and hyperglycemia as factors that can independently regulate HSPC function and/or alter the BM niche, influencing HSPC proliferation and maturation.^50–52^ Hypercholesterolemia and hyperglycemia promote leukocytosis, particularly of neutrophils and monocytes.^51,53,54^ In addition, epigenetic mechanisms or metabolic memory may permanently alter the functionality and inflammatory status of HSPCs in diabetic patients, whose glucose levels are inadequately controlled.^55–57^

The concordance of the changes in circulating progenitors and leukocyte populations in diabetic mice and humans with diabetes strongly suggests that BM myelopoiesis bridges the innate immune response to metabolite oscillations. Supporting this view, a newer study in a mouse model of obesity demonstrated that chronically inflamed visceral adipose tissue, through enhanced production of S100A8/A9, can signal to BM HSPCs to proliferate, expand, and increase the production of myeloid cells.^58^ The authors showed that in adipose tissue macrophages (ATM), S100A8/A9 induces TLR4/MyD88- and NLRP3 inflammasome-dependent production of IL-1β, which then travels to the BM to induce the proliferation of hematopoietic progenitor cells via IL-1R, ultimately resulting in monocytosis and neutrophilia.^58^ These studies suggest that obesity may have life-long effects on inflammatory responses by altering HSPC function and are consistent with the observations that adipose tissue inflammation and fibrosis are not resolved even after returning to normal weight upon weight loss.^15,16^ Increasing evidence notes that a western diet and lifestyle, as well as aging, drive the pandemic of chronic noninfectious degenerative diseases, termed “civilization diseases” (i.e., metabolic syndrome, cancer, and autoimmune and neurodegenerative diseases), and dramatically increase the susceptibility to and severity of infectious pandemics, such as the current COVID-19 pandemic.^59^ Therefore, a better understanding of the immunometabolic signaling networks that coordinate immune responses to environmental cues is warranted, with the ultimate goal of identifying new therapeutic strategies.

## Cancer metabolism and transcriptional control of emergency myelopoiesis

In stress/pathological conditions (e.g., infection and cancer), signals derived from the HSC niche modify the magnitude and composition of the hematopoietic output, a feature of immune regulation defined as “emergency” hematopoiesis, to guarantee a proper supply of both lymphoid and myeloid cells in response to increased demand.^60,61^ Most of the transcriptional mechanisms that guide dysfunctional myelopoiesis in cancer patients have been proven to be mechanistically linked to cancer-driven and/or preexisting metabolic reprogramming of the host. Acquisition of a tumor-supporting myeloid phenotype is the last event of a multistep process, encompassing initial immunometabolic reprogramming of MPs in the BM and later steps of terminal differentiation in the tumor microenvironment (TME), that occurs through modulation of selected transcriptional activities.^60^

### Role of orphan nuclear receptors (NRs) in metabolism-driven myelopoiesis

Significant advances have been made in elucidating the molecular mechanisms underlying the complex crosstalk between inflammation and metabolism and the emergent role of ligand-activated transcriptional regulators belonging to the NR superfamily,^62^ highlighting nutrient availability and intermediate metabolites as the main orchestrators of stem cell behavior. A number of studies have demonstrated a role of peroxisome proliferator-activated receptors (PPARs) in controlling HSC specification and functional polarization of myeloid cells through fine tuning of glucose and lipid metabolism,^63,64^ implicating a metabolism-centric regulation of lineage commitment.

We have demonstrated for the first time that myeloid-specific expression of the retinoic acid-related orphan NR (RORC1/RORγ) marks advanced cancer inflammation and that expansion of circulating RORγ^+^ myeloid cells is associated with an increased number of both immature suppressive cells (MDSCs) and TAMs.^65^ Ablation of RORγ in myeloid cells reprograms cancer myelopoiesis in favor of effector APCs capable of inducing potent antitumor CD4^+^ and CD8^+^ T cell responses and tumor regression.^65^ Interestingly, cholesterol precursors (i.e., desmosterol) and oxysterols are known to be potent endogenous RORγ agonists,^66,67^ and RORγ has been shown to be an important player in the circadian regulation of lipid and cholesterol metabolism.^68^ In addition, RORγ has a key role in adipocyte differentiation and mediating insulin sensitivity^10^ and is upregulated in patients with severe obesity.^69^ Altogether, these studies suggest that myelopoiesis and myeloid lineage fate are tightly regulated by circadian oscillations in metabolites. Therefore, profound changes in dietary lipid composition and insulin sensitivity may contribute to pathologic dysregulation of myelopoiesis and are one plausible mechanistic link between cancer and obesity.

### Role of myeloid transcription factors in metabolism-driven myelopoiesis

Additional transcriptional mechanisms contribute to the metabolic plasticity of myelopoiesis. CCAAT enhancer-binding protein-α (C/EBPα) is a major regulator of “steady state” granulopoiesis^70^ that interacts with the p50 NF-κB subunit to stimulate neutrophil production during acute inflammation.^71^ Of note, the role of p50 NF-κB in the functional diversity of myeloid cells has been delineated, demonstrating that its nuclear accumulation, in response to tumor-derived prostaglandin E2 (PGE2), promotes the suppressive phenotype of MDSCs and limits the antitumor efficacy of ICIs.^72^ A recent study demonstrated that inhibition of the NF-κB family member c-Rel transforms tumor-promoting MDSCs into effector APCs, resulting in inhibition of CD4^+^CD25^+^ Treg cell expansion and consequent activation of potent antitumor T cell responses, through metabolic reprogramming via C/EBPβ.^73^ In contrast with C/EBPα, C/EBPβ ^74^, and signal transducer and activator of transcription 3 (STAT3)^75^ play major roles in emergency conditions, contributing to MDSC accumulation.^76^ Importantly, C/EBPβ accumulation contributes to β cell failure and IR in mice by enhancing susceptibility to ER stress^77^ and is crucial in regulating diet-induced inflammation and hyperlipidemia in hematopoietic cells and macrophages.^78^ Of relevance, C/EBPβ expression is controlled by RORγ in cancer-associated myeloid cells,^65^ thus indicating a RORγ-C/EBPβ axis as a novel integrator of cancer myelopoiesis and lipid homeostasis. These pioneering studies suggest that the myeloid transcriptome and coordination of immune responses by myeloid cells are fine-tuned to stress and environmental cues through metabolic reprogramming.

### NAD metabolism in myeloid cell mobilization and differentiation

**Fig. 1:**
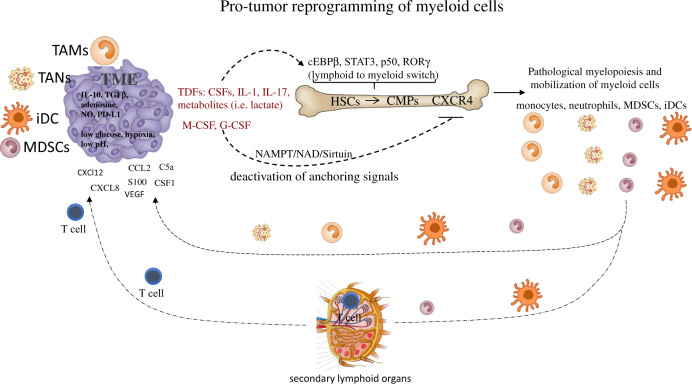
Protumor reprogramming of myeloid cells. Tumor-derived factors (TDFs), including cytokines, myeloid growth factors and metabolites, induce transcriptional activities (i.e., expression of cEBPβ, STAT3, p50, and RORC1/RORγ) guiding the enhanced proliferation and lymphoid to myeloid switch of HSCs. In parallel, activation of CSF-dependent induction of iNAMPT provides enhanced NAD-dependent activation of the sirtuin 1 deacetylase, which inhibits the HIF-1α-dependent and p65 NF-κB-dependent transcription of CXCR4. Deactivation of the anchoring signal CXCR4 mobilizes myeloid cells from the bone marrow, allowing peripheral expansion of myeloid populations (monocytes, neutrophils, MDSCs, and DCs). These cells reach the tumor site through the circulation and infiltrate the tumor tissue in response to tumor-derived chemotactic signals (TDCFs) (i.e., CXCL2, CXCL8, CCL2, S100, VEGF, C5a, and CSF1). In particular, DCs and MDSCs enter the secondary lymphoid organs (lymph nodes and spleen), eliciting inhibitory signals to T cells. Once in the tumor, myeloid cells undergo a further step of reprogramming in response to inhibitory molecules (IL-10, TGFβ, adenosine, NO, and PD-L1) and microenvironmental conditions (low glucose levels, hypoxia, and low pH), terminally differentiating into myeloid suppressor cells (TAMs, TANs, MDSCs, and immature DCs). Overall, the tumor-dependent reprogramming of myeloid populations has to be considered a multistep program, which comprises induction of emergency myelopoiesis (enhanced proliferation and the “lymphoid to myeloid” switch), mobilization to the periphery and final intratumor reprogramming. Common myeloid precursors (CMPs), hematopoietic stem cells (HSCs), tumor-derived factors (TDFs), immature DCs (iDCs)

Cancer cells display an atypical metabolic balance featuring increased glucose uptake and fermentation of glucose to lactate, even in the presence of oxygen and functioning mitochondria.^79^ This metabolic setting influences the crosstalk between tumor cells and tumor-infiltrating immune cells, creating competition for essential nutrients (glucose, in particular) and immunosuppression, which consequently hinder the therapeutic efficacy of anticancer immunotherapy.^80^ TME metabolism requires the cofactor nicotinamide adenine dinucleotide (NAD), which functions in many critical redox processes necessary for cancer cells and immune cells.^81^ Based on this, inhibitors of intracellular nicotinamide phosphoribosyltransferase (iNAMPT), the rate-limiting enzyme of NAD production in its salvage pathway, have entered clinical trials for solid and nonsolid tumors due to their ability to lower NAD and ATP levels and interfere with malignant cell growth.^82^ We recently reported that M-CSF, in addition to inducing PU.1-driven myeloid differentiation, has a direct role in controlling iNAMPT activity. Elevated iNAMPT in MPs causes negative regulation of the CXCR4 retention axis of hematopoietic cells in the BM,^83^ thus disengaging these cells and allowing the mobilization of suppressor myeloid cells to the periphery. In agreement, iNAMPT inhibition prevents MDSC mobilization, reactivates specific antitumor immunity, and enhances the antitumor activity of ICIs^83^ (Fig. 1).

The system composed of iNAMPT and the NAD-dependent protein deacetylase sirtuin 1 (SIRT1) plays a key role both in maintaining correct energy metabolism and in enhancing the robustness of physiological processes that control the resolution of the inflammatory response.^83,84^ In this regard, emerging evidence shows an age-related loss of iNAMPT/NAD^+^/SIRT1 activity that undermines antioxidant, metabolic and anti-inflammatory systems.^85,86^ Furthermore, iNAMPT activity is necessary for the differentiation of anti-inflammatory myeloid cells under stress conditions.^83^ NAMPT converts nicotinamide into nicotinamide mononucleotide (NMN), a precursor of NAD,^87^ which is actively consumed by NAD-dependent enzymes (e.g., sirtuins, mono-, and poly-(ADP-ribose) polymerases/ARTs and CD38/CD157)^84^ that control a variety of metabolic and stress responses through modulation of distinct transcriptional activities. In particular, the deacetylase SIRT1 acts homeostatically by repressing the transcription of HIF-1α-^88^ and p65 NF-κB-dependent genes.^89^ Of relevance, SIRT1 was shown to promote alternative/M2 macrophage polarization^83,90^ and suppressive activity of MDSCs.^91^ Of note, C/EBPα and C/EBPβ are controlled by NAD metabolism,^92^ indicating that in addition to insulin, glucose and lipids, regulators of cellular redox reactions may play a key role in myeloid homeostasis.

NAMPT expression is increased in various diseases, including chronic inflammatory conditions (e.g., rheumatoid arthritis), metabolic diseases (e.g., diabetes), and cancer.^91^ The robustness of the NAMPT/NAD^+^/SIRT1 system is controlled by the nutritional supply of tryptophan, and tryptophan and vitamin B3 (niacin) enable the primary and rescue pathways for the synthesis of NAD, respectively.^87^ Furthermore, administration of vitamin D3 to tumor-bearing mice (bearing metastatic Lewis lung carcinoma) reduces tumor-induced suppressor myeloid cells and enhances T cell functions^93^ and the differentiation of CD34^+^ immature myeloid cells.^94^ Importantly, recent studies have associated increased levels of extracellular NAMPT/visfatin with overweight/obesity, type 2 diabetes mellitus, IR, metabolic syndrome, and cardiovascular disease.^95^ Therefore, a better understanding of obesity-induced alterations in redox homeostasis and oxidative stress may provide us with important novel immunotherapeutic targets to reprogram pathogenic myelopoiesis in favor of effector APCs capable of coordinating potent antitumor responses.

## Interactions between metabolism and myeloid cell activation state

Of relevance, myeloid cells, macrophages in particular, express different functional programs in response to microenvironmental signals, a property defined as plasticity. The extremes of this functional spectrum are generally defined as classical (M1) and alternative (M2) activation states, which identify a cytotoxic and inflammatory phenotype as opposed to an anti-inflammatory and immunosuppressive phenotype, respectively.^96^ The shift between these antithetical activation states plays a distinct role in disease and has inspired new anticancer strategies aimed at the functional reprogramming of myeloid cells.^96^ Notably, evidence indicates that selected metabolic pathways can alter immune cell differentiation and direct effector functions.^97^ In response to M1-polarizing signals (i.e., LPS and IFNγ), macrophages fuel their energy requirements by enhanced glycolysis, associated with high flux through the pentose phosphate pathway, FA synthesis and a truncated tricarboxylic acid cycle, leading to accumulation of succinate and citrate.^98^ This metabolic profile induces the activation of transcriptional factors (i.e., NF-κB and STAT1) supporting the expression of a pro-inflammatory and antimicrobial program.^96^ Conversely, in response to IL-4, M2-polarized macrophages obtain energy through oxidative phosphorylation (OXPHOS)^99^ and fatty acid oxidation (FAO). In agreement, STAT6-dependent upregulation of the transcription factors peroxisome PPAR γ-coactivator-1β, PPARγ, and PPARδ drives the expression of genes that are crucial promoters of both oxidative metabolism and anti-inflammatory activities.^100–102^

In addition to macrophages, DCs and neutrophils are strongly affected by metabolic cues. DCs are the most potent antigen-presenting cells (APCs) of the immune system, and their phenotype is influenced by inflammatory and metabolic disorders.^103^ Tumors alter host hematopoiesis and induce an immature DC phenotype with immune-suppressive properties. In particular, cancer cell-derived immune-suppressive factors (VEGF, IL-10, and PGE2) disable DC differentiation, maturation, migration, and functions.^104^ Furthermore, lipid and amino acid metabolism modulate DC functions. Of relevance, while 27 hydroxycholesterol acts on HSCs via estrogen receptor α to increase their proliferation and mobilization,^105^ oxysterols, which are produced through enzymatic and nonenzymatic oxidation of cholesterol,^106^ interact with liver X receptors exerting an anti-inflammatory role on DCs.^107^ In agreement, oxysterols produced by tumor cells impair antigen presentation by inhibiting CCR7 expression on DCs.^108^ DC functionality can also be compromised by the uptake of lipids enriched within the TME, which lead to DC dysfunctions.^109^ Furthermore, DC immunogenicity is hampered by both TAMs and MDSCs through the production of copious amounts of indoleamine 2,3-dioxygenase 1 (IDO1), which converts tryptophan into kynurenines (Kyns).^110^ The metabolic plasticity of DCs is particularly evident during their progression from an immature to a mature state. Immature DCs generate ATP through OXPHOS, a process primarily driven by FAO.^111^ In response to maturation signals, DCs undergo a metabolic shift towards glycolysis.^112^ DC commitment to glycolysis correlates with activation of HIF-1, which is necessary to enhance the expression of glycolytic enzymes.^113^ Nevertheless, several tumors actively inhibit glycolysis and lipid synthesis in these cells, exacerbating impaired metabolic and immunologic functions of DC.^114^

The disrupted metabolic flux of cancer cells, characterized by aerobic glycolysis (Warburg effect), results in the preferential conversion of pyruvate to lactate, which in turn impairs the maturation of DCs^115^ and M2 polarization of TAMs.^116^ Adenosine production by tumors is an additional mechanism that impairs DC antitumor activities.^117^ Mechanistically, adenosine promotes the accumulation of cAMP in DCs and the consequent activation of the PKA and Epac pathways that polarize these cells to a tumor-promoting phenotype (IL-10^high^/IL-12^low^).^118^ Notably, the antitumor activity of CT has also been attributed to the induction of “immunogenic cancer-cell death”, which favors the processing and presentation of dead cell-associated antigens by DCs.^119^

Neutrophils have a short half-life of 7–10 h in both humans and mice.^120^ In accordance with their high turnover rates and rapid adaptability to diverse conditions of the tissue microenvironment, a number of studies have demonstrated their functional plasticity and capacity to extend their life in response to cytokines (i.e., IL-1β, IL-6, and TNF) and granulocyte colony-stimulating factor (G-CSF).^121,122^ Tumor-associated neutrophils (TANs) can elicit cytotoxic activity against tumor cells and promote inhibition of metastasis.^123^ However, contrasting evidence suggests that TANs can support tumor angiogenesis, cancer cell migration and invasion and immunosuppression.^120^ According to these results, IL-1β-driven IL-17 expression by γδ T cells promoted TAN expansion in mammary cancer-bearing mice, which eventually suppressed cytotoxic CD8^+^ T cell functions.^124^ Furthermore, PD-L1^+^ neutrophils from patients with hepatocellular carcinoma effectively suppressed the proliferation and activation of CD4^+^ and CD8^+^ T cells via PD-1/PD-L1 interactions.^125^ Of relevance, a high neutrophil-to-lymphocyte ratio (NLR) correlates with poor clinical outcome in patients with advanced-stage cancers,^126^ and a recent meta-analysis on 39 different malignancies identified tumor-infiltrating neutrophils as the immune population associated with the worst prognosis.^127^ In line with these contrasting observations, a recent paradigm has highlighted the phenotypic plasticity of TANs, which in response to microenvironmental signals can either display an inflammatory (N1) or an alternative (N2), tumor-promoting activation state. Transforming growth factor-β (TGFβ) was demonstrated to promote the N2 phenotype, whereas interferon-β leads to an antitumor (or N1) phenotype.^128^ Neutrophils are highly dependent on HIF-1 regulation, and in general, hypoxic conditions increase neutrophil inflammatory functions,^129,130^ promoting tissue infiltration, survival, activation, and cytokine release. In agreement, evidence indicates that neutrophils can suppress CTLs via HIF-1α-dependent^131^ iNOS-mediated nitric oxide (NO) production and reactive oxygen species (ROS).^124^

### Amino acid metabolism and suppressor myeloid cells

Consumption of essential amino acids is a classic example of how tumors exploit metabolic pathways to generate molecules endowed with immunomodulatory activities and deplete nutrients essential for T cells. In particular, TAMs and MDSCs express high levels of IDO1, an enzyme that converts tryptophan into its immunosuppressive catabolite Kyn, which is capable of inducing the expansion of regulatory T (Treg) cells,^80^ depriving T cells of an essential nutrient^132^ and hindering the immunogenicity of DCs.^110^ l-arginine depletion is one of the main mechanisms by which MDSCs inhibit antitumoral T cell activity; however, granulocytic (PMN-MDSCs) and monocytic (M-MDSCs) subsets of MDSCs use distinct enzymes or arginine metabolism to generate immunosuppression. In particular, l-arginine depletion by ARG1 predominantly occurs in PMN-MDSCs and leads to downregulation of T cell receptor (TCR)-ζ chain expression and inhibition of the cyclin-dependent kinase pathway regulating the cell cycle,^133^ as well as downregulation of the ornithine and polyamine generation that supports tumor cell proliferation.^134^ In contrast, M-MDSCs utilize the nitric oxide synthase (iNOS) enzyme to produce NO and promote tyrosine nitration and S-cysteine nitrosation of various proteins.^135,136^ Several lines of evidence also indicate that both enzymes can contribute to the immunosuppressive activities of human MDSCs.^1^

Cysteine represents an additional essential nutrient for T cells that is introduced from the extracellular space. In contrast with APCs that import extracellular oxidized cystine and export the cysteine used by T cells, MDSCs only uptake cystine (using the AA antiporter xc), thus limiting the extracellular pool of cysteine required for T cell activation.^137^

## Host insulin metabolism and cancer progression

In the early 1990s, the role of TNF-α as a pro-inflammatory cytokine linked to IR was demonstrated in the adipose tissue of obese mice.^138^ Later, studies in healthy and obese subjects confirmed that pro-inflammatory cytokines and immune cell infiltration were involved in glucose homeostasis.^139^ These findings raised interest in the potential role of IR in obesity-induced chronic inflammation, which is now known to be associated with cancer development and aggressiveness.^140^ The relationship between insulin, insulin sensitivity, the insulin growth factor (IGF) receptor family and cancer is now well established. Insulin and insulin-like growth factor 1 (IGF-1) activate insulin receptor and IGF-1 receptor, respectively, which are expressed at higher levels in malignant cells and support their proliferation.^141^ In addition, both insulin and IGF-1 increase sex hormone synthesis and reduce sex hormone-binding globulin levels, leading to elevated levels of estrogens and other endocrine tumor promoters.^141^ Moreover, obesity itself has been hypothesized to adversely impact the response to CT, not only through metabolic perturbations and underlying IR, adipokine production, and the IGF-1 axis but also by affecting drug delivery, pharmacokinetics, and transport.^142^ Of relevance, the obesity-driven transition of the macrophage activation state from “M2-like” to “M1-like” promotes inflammation and potentially contributes to IR.^143^ Macrophages are very prominent in adipose tissue, where they can reach proportions of up to 50% of all cells.^144^ Within breast adipose tissue, obesity leads to chronic, macrophage-driven inflammation, suggesting that obese breast cancer patients may benefit from metabolic targeting.^145^ In general, the inflammatory state induced by neoplastic processes might increase the cancer cell proliferation and paracrine-related effects mediated by inflammatory cytokines, such as increased angiogenesis^146^ and myeloid cell accumulation (TAMs and MDSCs), which orchestrate the creation of an immunosuppressive environment.^147^

Epidemiological observations have provided evidence that higher circulating insulin levels are associated with an adverse outcome in early BC patients.^148,149^ From this perspective, the insulin pathway may represent a therapeutic target, especially in patients with high plasma insulin levels. Hyperinsulinemia may reflect an altered level of insulin sensitivity and be associated with chronic inflammation, characterized by high levels of IL-6, C-reactive protein, tumor necrosis factor-α, fibrinogen, and the cell adhesion molecules ICAM-1 and VCAM-1.^150^ Moreover, hyperinsulinemia induces proliferative tissue abnormalities due to strong anabolic effects, resulting in the enhancement of DNA synthesis and cell proliferation. In women with early BC without diabetes, it has been observed that hyperinsulinemia is associated with the presence of IR.^151^ In our recent publication, we hypothesized that insulin might exert its influence on tumor aggressiveness by modulating gene expression at the level of breast cancer cells. In particular, by using publicly available gene datasets, we recently identified a gene signature based on the differential expression of 15 genes related to the insulin (27%), chronic inflammation (30%), and IGF pathways (40%) that was strongly associated with disease-free survival in early breast cancer.^152^ These data suggest that it is possible to identify a subset of BC patients whose prognosis is modulated by a set of genes related to the insulin pathway.

From this perspective, the potential antitumor effect of the antidiabetic drug metformin has been extensively studied due to its antiproliferative activity in vitro.^153,154^ In cancer patients, however, metabolic targeting does not translate into a measurable clinical benefit, probably due to the complexity of the IGF-1R/insulin receptor system and the presence of parallel pathways of growth and survival, as well as the lack of appropriate patient selection markers.^154,155^ Moreover, a number of clinical studies evaluating anti-IGF drugs (either monoclonal antibodies or TKIs) reported inconsistent results or were complicated by excessive metabolic toxicity (hyperglycemia). We recently performed a translational study to evaluate the potential role of IGF-1R expression on circulating tumor cells (CTCs) of patients enrolled in the MYME trial, which compares first-line CT with first-line CT plus metformin in HER2-negative, metastatic BC patients without diabetes. Our data demonstrate that patients with loss of IGF-1R on CTCs have a significantly worse outcome than patients with IGF-1R expression on CTCs, providing a possible clue for improved patient stratification strategies aimed at metabolic targeting in advanced BC.^152^ Overall, the relationship between IR, metabolic impairment, the underlying chronic inflammatory status and cancer needs to be extensively evaluated to better develop a strategy for “metabolic targeting”.

Additional studies addressing the impact of obesity and IR on the outcome of ICI therapy have provided controversial data to date. ICIs have been reported to cause immune-mediated damage to islet cells, leading to ICI-induced type 1 diabetes mellitus (T1DM).^156^ On the other hand, there are a number of reports supporting an “obesity paradox,” in which patients with higher body mass index, i.e., patients with overweight or obesity, have an improved outcome if treated with immunotherapy for advanced tumors,^157^ indicating a close connection between the immune system and patient metabolic status. Stratifying a cohort of patients with metastatic melanoma into groups based on receipt of first-line immunotherapy revealed a moderate but insignificant association between overweight or obesity and better progression-free survival in patients who received first-line immunotherapy. Conversely, an association with worse progression-free survival was observed in patients who received non-first-line ICIs.^158^ Therefore, studies that better depict the contribution of IR and obesity to the outcome of ICI therapy are needed to adapt patient care to metabolic status.

### Tumors alter the glycolysis vs. OXPHOS balance of myeloid cells to control their activation state

Recent studies indicate that impaired OXPHOS accompanied by increased glycolysis may be a significant contributor to increased myelopoiesis and heightened myeloid cell activation under acute and chronic settings.^159,160^ Enhanced tumor glycolytic flux converts a major fraction of pyruvate into lactate, even under normoxic conditions (Warburg effect). The conversion of pyruvate into lactate is mediated by lactate dehydrogenase A, a hypoxia-inducible factor 1α (HIF-1α) target.^161,162^ Lactate-mediated inhibition of DC maturation^115^ is paralleled by the generation of MDSCs.^162^ Moreover, a recent study suggests that lactate operates as an endogenous inhibitor of histone deacetylases,^163^ transcriptionally regulating a number of genes that not only are involved in metabolism and transcriptional control but also participate in immune response fate. Furthermore, increased tumor glycolysis enhances GM-CSF, G-CSF, C/EBPβ^164^, and NF-κB expression,^165^ which represent major immune signaling pathways that support MDSC differentiation.

In addition to the competition for glucose and essential metabolites in inflamed tissue, intracellular metabolic aberrations may reprogram immune cell fate, survival and function. A new study shows that tumor MDSCs suppress glycolysis-mediated T cell effector functions by transferring methylglyoxal to CD8^+^ T cells via direct cell contact. The acquisition of methylglyoxal by T cells coincides with a reduction in free l-arginine and a concomitant increase in the products of glycation reactions between methylglyoxal and l-arginine, suggesting that depletion of l-arginine in the cytosol paralyzes T cell functions.^166^ Under homeostatic conditions, cells are protected against methylglyoxal toxicity by different mechanisms, particularly the glyoxalase system, which represents the most important pathway for the detoxification of methylglyoxal.^167^ Methylglyoxal is formed as a byproduct of glycolysis and is a major cell-permeant precursor of advanced glycation end-products (AGEs), while engagement of RAGE, the receptor for AGEs, is shown to activate downstream signaling and evoke oxidative stress and inflammation in diabetes.^168^ Changes in the intracellular levels of glycolytic metabolites are linked to the inflammatory phenotype of immune cells implicated in autoimmune disorders, such as systemic lupus erythematosus, rheumatoid arthritis, multiple sclerosis, and diabetes. Notably, targeting metabolic effectors, such as targeting of mTOR by rapamycin, hexokinase by 2-deoxy-D-glucose, and adenosine monophosphate (AMP)-activated protein kinase by metformin, may be used to ameliorate autoimmune inflammation.^169,170^ Preliminary studies suggest that extrinsic and intrinsic increases in glycolysis and uncoupling from OXPHOS may lead to aberrant myeloid cell differentiation, expansion, and activation in a broad range of inflammatory diseases.

## Interplay between cancer therapy, metabolism, and myeloid suppressor cells

Deregulated myelopoiesis sustains malignant transformation and progression by shaping the TME via interactions with tumor cells, stroma, and other infiltrating immune cells, ultimately promoting cell growth, angiogenesis, and diversion and skewing of the adaptive immune response. The generation of suppressor myeloid cells is a driving force for tumor progression and therefore a promising therapeutic target. In addition, available anticancer strategies feature myeloid-specific activities as part of their antitumor actions. In particular, modulation of MDSCs is now accredited as a key therapeutic strategy due to the tumor-promoting phenotype of MDSCs and their capacity to affect the efficacy of CT, radiotherapy, and immunotherapy.

### Metabolic effects of cancer therapy

Newer studies have shown that cancer treatment-induced metabolic syndrome (CTIMetS) is an especially prevalent and harmful side effect of CT. Long-term survivors of childhood, breast, colorectal and testicular cancer, and of several hematological malignancies face an increased risk of treatment-induced cardiovascular disease^171^ and metabolic syndrome.^172^ Metabolic comorbidities may adversely affect patient survival and quality of life and might be an important link between cancer treatment cardiovascular toxicity and accelerated atherosclerosis in cancer survivors.^173^ Obesity is a contributing factor to the higher occurrence of metabolic syndrome and cardiovascular morbidity in cancer survivors.^174^ In addition, IR may be driven by CT. Alkylators, anthracyclines, camptothecins (e.g., irinotecan), epipodophyllotoxins (e.g., etoposide), and platinum-based treatments may drive IR due to mitochondrial dysfunction through increased production of ROS.^175^ Antimetabolites such as capecitabine can decrease hepatic lipid export, causing steatosis, which is associated with decreased insulin sensitivity.^176^ Furthermore, anemia, which is a common side effect of anticancer treatments, may cause adipose tissue hypoxia, leading to macrophage activation and inflammatory cytokine release.^175^ Therefore, CT contributes to the development of CTIMetS mostly through weight gain but may also indirectly affect other metabolic syndrome components, such as dyslipidemia or IR.

Although the etiology of metabolic syndrome in noncancer patients probably differs from the etiology in cancer patients treated with conventional cancer therapy,^172^ the same treatment strategies to restore metabolic homeostasis may have similar positive effects on the prevention and treatment of the different components of metabolic syndrome and improvement of life quality and life expectancy.

Interestingly, in premenopausal and postmenopausal early breast cancer patients with no preexisting metabolic syndrome, adjuvant, or neoadjuvant CT was associated with an increased prevalence of metabolic syndrome and related anthropometrics, biomarkers of glucose metabolism, and inflammation.^177^ Identifying markers able to capture the complex interplay among host metabolism, myelopoiesis, CT, and the TME might contribute to integrating preclinical and clinical research.

Cranial and abdominal radiotherapy are major risk factors for obesity, dyslipidemia, and IR in childhood cancer survivors.^178,179^ The pancreas has always been considered relatively insensitive to radiation.^180^ However, recent evidence suggests that radiation may induce apoptosis of pancreatic beta cells and consequently decrease insulin production, leading to hyperglycemia, elevated FFA levels, hypertriglyceridemia, and IR.^179,181^ Deficiency of growth hormone is the most common endocrine dysfunction in patients treated with cranial radiotherapy and is associated with obesity, dyslipidemia, and IR.^182^ Growth hormone contributes to lipolysis and has an insulin-like influence because of its relationship with the production of IGF-1, which results in glucose uptake.^172^ Preliminary data suggest that assessment of metabolic fitness and myeloid function may be critical to avoid iRAEs and to increase the efficacy of conventional cancer therapy. MDSC levels may be used as a novel biomarker for metabolic syndrome and related immune dysfunction, as MDSCs are the end product of profound cellular metabolic changes.

### Chemotherapy (CT) and MDSCs

CT is a long-standing inclusion in the therapeutic armamentarium against cancer. Mounting evidence from preclinical studies has revealed the contribution of the host immune system to the efficacy of several anticancer drugs. Most preclinical studies support CT-induced inflammation as a mechanism to reinforce aberrant myelopoiesis, which serves as a counterregulatory adaptation to prevent unnecessary damage from chemical insult.^183^ Enhancement of MDSC suppressive activity is described with doxorubicin and with high-dose cyclophosphamide, among other treatments.^184^ In contrast, other preclinical data have shown that a number of cytotoxic agents, including docetaxel, 5-fluorouracil, and gemcitabine, can induce MDSC apoptosis.^185–187^ Treatment with cyclophosphamide can be considered a prime example of the complexity of the interplay between CT and the immune system, since both immunostimulating effects and the induction of immunosuppressive cells have been described with this agent.^188–192^

In breast cancer, data from clinical studies prospectively evaluating the effects of CT on MDSCs are scant, and the results are somewhat conflicting.^193–195^ However, these data are far from conclusive, considering the limited sample sizes, the heterogeneity of the patient populations (in terms of breast cancer phenotype classified according to hormone receptor and HER2 expression), as well as differences in G-CSF use, which is one of the key drivers of aberrant expansion of myeloid cells.

In patients with advanced pancreatic cancer, MDSCs are significantly elevated compared to the numbers in healthy controls.^196^ In a pilot nonrandomized trial, MDSCs were significantly decreased in patients treated with the combination of gemcitabine and omega 3 fatty acid, while no significant change was observed in patients treated with gemcitabine alone. Intriguingly, better progression-free survival was reported for patients treated with the combination. However, these findings should be interpreted with caution due to the nonrandomized nature of the study.^197^

In patients with nonmetastatic urothelial cancer of the bladder undergoing radical cystectomy, the percentages of total MDSCs and PMN-MDSCs in PBMCs were significantly lower in patients achieving a pathologic complete response than patients showing no response. Higher levels of MDSCs before surgery were also associated with worse overall survival.^198^

In patients with non-small-cell lung cancer receiving first-line platinum-based CT, a significantly worse outcome was reported for patients with higher M-MDSCs than for those with lower M-MDSCs. However, dynamic changes in MDSCs during CT were not evaluated.^199^ Overall, these data suggest that CT can impact the TME by promoting an antitumor immune response or by inducing MDSCs that counterregulate the immune response.

### Myeloid immunometabolism and immunotherapy

CT and radiotherapy still represent fundamental strategies in anticancer treatment. Nevertheless, in the past few years, restoring the immune response with ICIs has emerged as an effective strategy across different cancer types. Interestingly, though apparently counterproductive from a theoretical standpoint, the combination of these two strategies has resulted in clinically meaningful results.^200–203^ However, the magnitude of clinical benefit with immunotherapy is heterogeneous, since a significant proportion of patients do not respond or even experience hyperprogression.^204^ In this context, circulating immune-related biomarkers are particularly attractive. Cancer mortality is almost doubled in patients with elevated MDSCs.^205^ It has been reported that the presence of circulating MDSCs predicts higher stage and worse survival rates^206^ and increases the risk of resistance to ICIs.^207^ Measuring MDSCs is a novel and yet-to-be-exploited strategy for treating cancer. MDSCs are difficult to detect and quantify because their phenotypic signature includes multiple surface markers studied by flow cytometry or immunohistochemistry, and those markers can detect immature myeloid cells but cannot predict suppressor function. A consensus phenotype of human MDSCs has recently emerged^208,209^ and can predict dysregulated myelopoiesis when evaluated together with clinical parameters.

The importance of MDSCs in promoting resistance to immunotherapy was not recognized until the first studies demonstrated that MDSCs have potent utility in inhibiting T cell and NK cell activity, contributing to resistance to immunotherapy and predicting resistance to ICIs.^210^ To date, the majority of the clinical data are available for melanoma patients treated with ipilimumab, in which a potential role of the frequency of monocytic MDSCs as a predictive marker of response has been suggested.^211^ In another study assessing the frequencies of MDSCs and Treg cells in 209 melanoma patients treated with ipilimumab, MDSC frequencies and CD4^+^CD25^+^FoxP3^+^ Treg cell frequencies were significantly associated with survival.^207^ In prostate cancer patients treated with ipilimumab combined with a cancer vaccine, a lower frequency of circulating MDSCs was found to correlate with an increased overall survival.^212^ Several ongoing trials of chemoimmunotherapy are prospectively evaluating MDSCs and TAMs, with the aim of elucidating the mechanisms underlying different patterns of response and different outcomes upon treatment. In line with these studies, the field of cancer immunotherapy has focused on developing therapeutic strategies to eliminate MDSCs.

Tumor-infiltrating lymphocytes (TILs) are critical for inducing tumor regression; however, TILs in patients with “cold” myeloid-driven tumors are not sufficient to overcome tumor-associated immunosuppression. It is becoming clear that eliminating inflammation-driven emergency myelopoiesis is critical for enabling improved T effector-APC crosstalk, recruitment of antitumor immune responses, and inhibition of tumor-promoting angiogenesis. Emerging studies support the view that targeting tumor metabolism in combination with immunotherapy enhances the efficacy of immunotherapy.

In mice, inhibition of FAO significantly decreases FA uptake and inhibits the immunosuppressive function of MDSCs at the tumor site in Lewis lung carcinoma (3LL).^40^ All-trans retinoic acid (ATRA), a metabolite of vitamin A, induces MDSCs to differentiate into APCs as well as myeloid maturation, which correlate with an improvement of the antitumor effector T cell response^213^ and reduced MDSC levels in tumor-bearing mice and tumor patients.^214,215^ ATRA affects MDSCs by upregulating the expression of glutathione through ERK1/2 activation to neutralize a large amount of ROS in MDSCs and promote MDSC differentiation.^216^

Inhibition of glucose uptake by a Glut1 inhibitor to inhibit exacerbated glycolysis in stroma cells, MPs and myeloid cells may provide a novel therapeutic approach to prevent myelopoiesis-driven inflammatory diseases.^217^ AMP-activated protein kinase (AMPK) activation can inhibit several major immune signaling pathways, e.g., the JAK-STAT, NF-κB, C/EBPβ, CHOP, and HIF-1α pathways. Activation of these pathways regulates cellular immunity in cooperation with pathways controlling energy metabolism, which favor the expansion and activation of MDSCs.^165^ Furthermore, a recent study by Strauss et al. showed that immune checkpoints such as PD-1 suppress the differentiation of MPs to effector APCs and promote the expansion of MDSCs through metabolic reprogramming of myeloid precursors.^218^ Metformin, a widely used drug in treating and curing type II diabetes, has been proven to reduce the incidence of cancers, reduce mortality, increase the response to radiotherapy and CT, optimize tumor cell migration, and reduce the likelihood of relapse.^219^ Metformin inhibits mTOR activity by activating ATM (ataxia telangiectasia mutated) and LKB1 (liver kinase B1) and AMP-activated kinase (AMPK), thus preventing protein synthesis and cell growth,^219^ as well as MDSC expansion.^220,221^ Taken together, these findings provide a rationale for combining strategies reprogramming the metabolism of MDSCs with immunotherapeutic strategies in cancer treatment and prevention.

Recent data have highlighted the crucial connection between metabolism and cancer immunotherapy. In particular, a new experimental glutamine antagonist has been shown to induce tumor regression not only through cancer cell starvation but also by activating effector T cells, thus dismantling the immunosuppressive TME. Indeed, T cells respond to glutamine antagonism by markedly upregulating oxidative metabolism and adopting a long-lived, highly activated phenotype. Exploiting different metabolic states of the components of the TME might contribute to improving the therapeutic armamentarium against cancer.^222^

## Concluding remarks

**Fig. 2:**
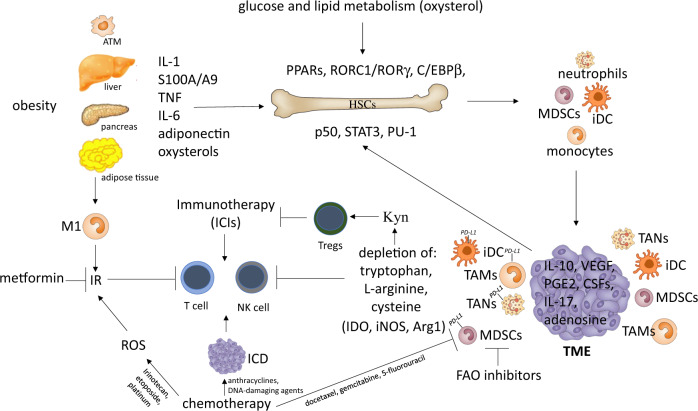
Interconnections between metabolism, cancer-related inflammation, myelopoiesis, and cancer therapy. Obesity and adipose tissue macrophages (ATMs) promote myeloid cell expansion by releasing various inflammatory cytokines and adipokines that activate selected transcriptional activities (PARs, RORC1/RORγ, and C/EBPβ) affecting HSC proliferation and differentiation. This myelopoietic boost is amplified by cancer cells that release additional myelopoietic factors, including CSFs, IL-1, IL-17, and PGE2. These factors induce myelopoiesis through the upregulation of specialized transcription factors (i.e., p50 NF-κB, STAT3, and PU-1). The production of adenosine, VEGF, and IL-10 by cancer cells induces the tumor-promoting phenotype (IL-10^high^/IL-12^low^) of iDCs. The emerging myeloid populations are then recruited to the tumor site, where they acquire suppressor phenotypes (TAMs, TANs, MDSCs, and iDCs) and establish an immunosuppressed tumor microenvironment (TME). The tumor site actively hinders the activation of T lymphocytes through the depletion of amino acids, orchestrated by both infiltrating myeloid suppressor cells and cancer cells that express immunosuppressive enzymes (IDO, iNOS, and Arg1). IDO activity, in particular, results in the production of the immunosuppressive catabolite kynurenine (Kyn), which is capable of inducing the expansion of regulatory T (Treg) cells. Further expression of immune checkpoint ligands (i.e., PD-L1) by myeloid suppressor cells contributes to the inhibition of antitumor immunity. The metabolic consequences of obesity also drive the transition of macrophages from “M2-like” to “M1-like” activation, contributing to inflammation-driven insulin resistance (IR). Of note, both obesity and select chemotherapeutics (i.e., irinotecan, etoposide, and platinum) can induce IR, interfere with the energetic balance and affect T cell activation. However, chemotherapy can also enhance antitumor immunity by promoting the immunogenic cell death (ICD) of cancer cells (i.e., anthracyclines, DNA-damaging agents) and by depleting MDSCs (i.e., docetaxel, gemcitabine, and 5-fluorouracil). In line with this, the inhibition of FAO significantly decreases FA uptake and inhibits the immunosuppressive function of MDSCs. Globally, the intersection of the host’s metabolic status, tumor metabolism, cancer inflammation and the quality of myelopoietic output strongly influences the response to therapy. ICD immunogenic cell death, IR insulin resistance, FAO fatty acid oxidation, FA fatty acid

The ability of the immune system as well as adipose tissue to expand and contract in response to fluctuations in nutrient availability is essential for the maintenance of whole-body homeostasis. Given the shortages of nutrients that mammals have faced for millions of years, the current programs involved in immune and adipose plasticity likely evolved to be highly efficient in promoting metabolic strategies to adapt to nutrient stress. Therefore, it is not surprising that many transcription networks critical for innate and adaptive immune cell functions are shared by adipose tissue and have a role in insulin signaling. Myelopoiesis, as the host first line of defense, requires very high plasticity and therefore shares many transcription and cytokine networks with adipocytes, and these networks provide MPs and myeloid cells with extra metabolites in response to environmental cues. Tumors perturb these adaptive networks by consuming oxygen and critical metabolites for immune and stromal cell function. In addition, these previously advantageous features may now represent a metabolic risk factor given the caloric excess of modern society. Acquisition of a tumor-promoting phenotype by myeloid cells as well as stress-triggered adipogenesis and IR are the results of a multistep process encompassing initial events originating in the BM and later steps operating in the TME.^60^ The interplay between inflammation and metabolism dictates transcriptional programs supporting the differentiation of myeloid suppressor cells (MDSCs, iDCs, and TAMs). These cells are being recognized as novel biomarkers for metabolism-compromised dysregulation of central tissue homeostasis, which leads to systemic immune dysfunction and persistent inflammation in cancer and metabolic syndrome-related inflammatory diseases (Fig. 2). Recent research highlights the potential therapeutic impact of targeting specific metabolic pathways and/or modifying the quantity and quality of myeloid output to stimulate anticancer immunosurveillance and prevent disease relapse. New studies are now required to carefully evaluate the myelopoietic and immunomodulatory impact of anticancer therapies, as well as their interplay with host immunometabolism. Immunometabolic characterization of the population of interest, cancer patients in particular, should therefore be sponsored, as it might establish new criteria for stratification of patients and therapeutic interventions. A lack of proactive and preventive efforts could lead to worldwide permeation of such immunometabolic dysfunctions, with an increased risk of developing resistance to and irAEs with immunotherapy and a consequent reduction in therapeutic options.

## References

[1] Gabrilovich DI, Ostrand-Rosenberg S, Bronte V (2012). Coordinated regulation of myeloid cells by tumours. Nat. Rev. Immunol..

[2] Weber R (2018). Myeloid-derived suppressor cells hinder the anti-cancer activity of immune checkpoint inhibitors. Front Immunol..

[3] Mantovani A, Marchesi F, Malesci A, Laghi L, Allavena P (2017). Tumour-associated macrophages as treatment targets in oncology. Nat. Rev. Clin. Oncol..

[4] Chavakis T, Mitroulis I, Hajishengallis G (2019). Hematopoietic progenitor cells as integrative hubs for adaptation to and fine-tuning of inflammation. Nat. Immunol..

[5] Escamilla-Tilch M (2013). The interplay between pathogen-associated and danger-associated molecular patterns: an inflammatory code in cancer?. Immunol. Cell Biol..

[6] Janeway CA (1989). Approaching the asymptote? Evolution and revolution in immunology. Cold Spring Harb. Symp. Quant. Biol..

[7] Pietras EM (2016). Chronic interleukin-1 exposure drives haematopoietic stem cells towards precocious myeloid differentiation at the expense of self-renewal. Nat. Cell Biol..

[8] Lin L (2012). Adipocyte expression of PU.1 transcription factor causes insulin resistance through upregulation of inflammatory cytokine gene expression and ROS production. Am. J. Physiol. Endocrinol. Metab..

[9] Eguchi J (2008). Interferon regulatory factors are transcriptional regulators of adipogenesis. Cell Metab..

[10] Meissburger B (2011). Adipogenesis and insulin sensitivity in obesity are regulated by retinoid-related orphan receptor gamma. EMBO Mol. Med..

[11] Pietras EM (2017). Inflammation: a key regulator of hematopoietic stem cell fate in health and disease. Blood.

[12] Liu Q (2020). Inhibition of PU.1 ameliorates metabolic dysfunction and non-alcoholic steatohepatitis. J. Hepatol..

[13] Natoli G, Ostuni R (2019). Adaptation and memory in immune responses. Nat. Immunol..

[14] Locati M, Curtale G, Mantovani A (2020). Diversity, mechanisms, and significance of macrophage plasticity. Annu Rev. Pathol..

[15] Divoux A (2010). Fibrosis in human adipose tissue: composition, distribution, and link with lipid metabolism and fat mass loss. Diabetes.

[16] Cancello R (2013). Permanence of molecular features of obesity in subcutaneous adipose tissue of ex-obese subjects. Int J. Obes..

[17] Casco S, Soto-Vega E (2016). Development of metabolic syndrome associated to cancer therapy: review. Horm. Cancer.

[18] Ramapriyan R (2019). Altered cancer metabolism in mechanisms of immunotherapy resistance. Pharmacol. Ther..

[19] Kreuzaler P, Panina Y, Segal J, Yuneva M (2020). Adapt and conquer: metabolic flexibility in cancer growth, invasion and evasion. Mol. Metab..

[20] Weiss JM (2020). The promise and peril of targeting cell metabolism for cancer therapy. Cancer Immunol. Immunother..

[21] Eckel RH, Grundy SM, Zimmet PZ (2005). The metabolic syndrome. Lancet.

[22] de Ferranti S, Mozaffarian D (2008). The perfect storm: obesity, adipocyte dysfunction, and metabolic consequences. Clin. Chem..

[23] Hotamisligil GS (2006). Inflammation and metabolic disorders. Nature.

[24] Vargas T (2014). Genes associated with metabolic syndrome predict disease-free survival in stage II colorectal cancer patients. A novel link between metabolic dysregulation and colorectal cancer. Mol. Oncol..

[25] You J (2015). Metabolic syndrome contributes to an increased recurrence risk of non-metastatic colorectal cancer. Oncotarget.

[26] Versini M, Jeandel PY, Rosenthal E, Shoenfeld Y (2014). Obesity in autoimmune diseases: not a passive bystander. Autoimmun. Rev..

[27] de Luca C, Olefsky JM (2008). Inflammation and insulin resistance. FEBS Lett..

[28] Horiguchi H (2018). Innate immunity in the persistent inflammation, immunosuppression, and catabolism syndrome and its implications for therapy. Front Immunol..

[29] Bernad A (1994). Interleukin-6 is required in vivo for the regulation of stem cells and committed progenitors of the hematopoietic system. Immunity.

[30] Patchen ML, MacVittie TJ, Williams JL, Schwartz GN, Souza LM (1991). Administration of interleukin-6 stimulates multilineage hematopoiesis and accelerates recovery from radiation-induced hematopoietic depression. Blood.

[31] Stroud CR (2019). Tocilizumab for the management of immune mediated adverse events secondary to PD-1 blockade. J. Oncol. Pharm. Pr..

[32] Mizuta H (2020). Hemophagocytic lymphohistiocytosis with advanced malignant melanoma accompanied by ipilimumab and nivolumab: a case report and literature review. Dermatol Ther..

[33] Hantel A, Gabster B, Cheng JX, Golomb H, Gajewski TF (2018). Severe hemophagocytic lymphohistiocytosis in a melanoma patient treated with ipilimumab + nivolumab. J. Immunother. Cancer.

[34] Singer K (2014). Diet-induced obesity promotes myelopoiesis in hematopoietic stem cells. Mol. Metab..

[35] Poitou C (2011). CD14dimCD16+ and CD14+CD16+ monocytes in obesity and during weight loss: relationships with fat mass and subclinical atherosclerosis. Arterioscler Thromb. Vasc. Biol..

[36] Friedrich K (2019). Perturbation of the monocyte compartment in human obesity. Front Immunol..

[37] Yarnell JW, Patterson CC, Sweetnam PM, Lowe GD (2004). Haemostatic/inflammatory markers predict 10-year risk of IHD at least as well as lipids: the Caerphilly collaborative studies. Eur. Heart J..

[38] Cannon CP, McCabe CH, Wilcox RG, Bentley JH, Braunwald E (2001). Association of white blood cell count with increased mortality in acute myocardial infarction and unstable angina pectoris. OPUS-TIMI 16 Investigators. Am. J. Cardiol..

[39] Calle EE, Rodriguez C, Walker-Thurmond K, Thun MJ (2003). Overweight, obesity, and mortality from cancer in a prospectively studied cohort of U.S. adults. N. Engl. J. Med..

[40] Hossain F (2015). Inhibition of fatty acid oxidation modulates immunosuppressive functions of myeloid-derived suppressor cells and enhances cancer therapies. Cancer Immunol. Res..

[41] Khadge S, Sharp JG, Thiele GM, McGuire TR, Talmadge JE (2020). Fatty acid mediators in the tumor microenvironment. Adv. Exp. Med. Biol..

[42] Hale M (2015). Obesity triggers enhanced MDSC accumulation in murine renal tumors via elevated local production of CCL2. PLoS One.

[43] Incio J (2016). Obesity-induced inflammation and desmoplasia promote pancreatic cancer progression and resistance to chemotherapy. Cancer Discov..

[44] Clements VK (2018). Frontline science: high fat diet and leptin promote tumor progression by inducing myeloid-derived suppressor cells. J. Leukoc. Biol..

[45] Bechtold M, Palmer J, Valtos J, Iasiello C, Sowers J (2006). Metabolic syndrome in the elderly. Curr. Diab. Rep..

[46] Bouchlaka MN (2013). Aging predisposes to acute inflammatory induced pathology after tumor immunotherapy. J. Exp. Med..

[47] Jiao Z (2013). Increased circulating myeloid-derived suppressor cells correlated negatively with Th17 cells in patients with rheumatoid arthritis. Scand. J. Rheumatol..

[48] Yin B (2010). Myeloid-derived suppressor cells prevent type 1 diabetes in murine models. J. Immunol..

[49] Amodio G (2019). Role of myeloid regulatory cells (MRCs) in maintaining tissue homeostasis and promoting tolerance in autoimmunity, inflammatory disease and transplantation. Cancer Immunol. Immunother..

[50] Swirski FK (2007). Ly-6Chi monocytes dominate hypercholesterolemia-associated monocytosis and give rise to macrophages in atheromata. J. Clin. Investig..

[51] Drechsler M, Megens RT, van Zandvoort M, Weber C, Soehnlein O (2010). Hyperlipidemia-triggered neutrophilia promotes early atherosclerosis. Circulation.

[52] Orlandi A (2010). Long-term diabetes impairs repopulation of hematopoietic progenitor cells and dysregulates the cytokine expression in the bone marrow microenvironment in mice. Basic Res Cardiol..

[53] Adler BJ, Kaushansky K, Rubin CT (2014). Obesity-driven disruption of haematopoiesis and the bone marrow niche. Nat. Rev. Endocrinol..

[54] Murphy AJ (2011). ApoE regulates hematopoietic stem cell proliferation, monocytosis, and monocyte accumulation in atherosclerotic lesions in mice. J. Clin. Invest.

[55] Giacco F, Brownlee M (2010). Oxidative stress and diabetic complications. Circ. Res..

[56] Berezin A (2016). Metabolic memory phenomenon in diabetes mellitus: achieving and perspectives. Diabetes Metab. Syndr..

[57] Giacco F (2015). GLP-1 cleavage product reverses persistent ROS generation after transient hyperglycemia by disrupting an ROS-generating feedback loop. Diabetes.

[58] Nagareddy PR (2014). Adipose tissue macrophages promote myelopoiesis and monocytosis in obesity. Cell Metab..

[59] Costa FF (2020). Metabolic syndrome and COVID-19: an update on the associated comorbidities and proposed therapies. Diabetes Metab. Syndr..

[60] Sica A, Guarneri V, Gennari A (2019). Myelopoiesis, metabolism and therapy: a crucial crossroads in cancer progression. Cell Stress.

[61] Pascutti MF, Erkelens MN, Nolte MA (2016). Impact of viral infections on hematopoiesis: from beneficial to detrimental effects on bone marrow output. Front. Immunol..

[62] Gerbal-Chaloin S, Iankova I, Maurel P, Daujat-Chavanieu M (2013). Nuclear receptors in the cross-talk of drug metabolism and inflammation. Drug Metab. Rev..

[63] Li G (2016). Hematopoietic knockdown of PPARdelta reduces atherosclerosis in LDLR-/- mice. Gene Ther..

[64] Vergori L (2015). PPARalpha regulates endothelial progenitor cell maturation and myeloid lineage differentiation through a NADPH oxidase-dependent mechanism in mice. Stem Cells.

[65] Strauss L (2015). RORC1 regulates tumor-promoting "Emergency" granulo-monocytopoiesis. Cancer Cell.

[66] Hu X (2015). Sterol metabolism controls T(H)17 differentiation by generating endogenous RORgamma agonists. Nat. Chem. Biol..

[67] Santori FR (2015). Identification of natural RORgamma ligands that regulate the development of lymphoid cells. Cell Metab..

[68] Takeda Y (2014). Retinoid acid-related orphan receptor gamma, RORgamma, participates in diurnal transcriptional regulation of lipid metabolic genes. Nucleic Acids Res.

[69] Ryden M (2016). The adipose transcriptional response to insulin is determined by obesity, not insulin sensitivity. Cell Rep..

[70] Hirai H (2006). C/EBPbeta is required for ‘emergency’ granulopoiesis. Nat. Immunol..

[71] Wang D, Paz-Priel I, Friedman AD (2009). NF-kappa B p50 regulates C/EBP alpha expression and inflammatory cytokine-induced neutrophil production. J. Immunol..

[72] Porta C (2020). Tumor-derived prostaglandin E2 promotes p50 NF-kappaB-dependent differentiation of monocytic MDSC. Cancer Res..

[73] Li X (2020). The c-Rel-c-Myc axis controls metabolism and proliferation of human T leukemia cells. Mol. Immunol..

[74] Akagi T (2008). Impaired response to GM-CSF and G-CSF, and enhanced apoptosis in C/EBPbeta-deficient hematopoietic cells. Blood.

[75] Zhang H (2010). STAT3 controls myeloid progenitor growth during emergency granulopoiesis. Blood.

[76] Marigo I (2010). Tumor-induced tolerance and immune suppression depend on the C/EBPbeta transcription factor. Immunity.

[77] Matsuda T (2010). Ablation of C/EBPbeta alleviates ER stress and pancreatic beta cell failure through the GRP78 chaperone in mice. J. Clin. Investig..

[78] Rahman SM (2016). C/EBPbeta in bone marrow is essential for diet induced inflammation, cholesterol balance, and atherosclerosis. Atherosclerosis.

[79] Warburg O (1956). On respiratory impairment in cancer cells. Science.

[80] Porta C (2018). Metabolic influence on the differentiation of suppressive myeloid cells in cancer. Carcinogenesis.

[81] Garten A (2015). Physiological and pathophysiological roles of NAMPT and NAD metabolism. Nat. Rev. Endocrinol..

[82] Galli U (2013). Medicinal chemistry of nicotinamide phosphoribosyltransferase (NAMPT) inhibitors. J. Med. Chem..

[83] Travelli C (2019). Nicotinamide phosphoribosyltransferase acts as a metabolic gate for mobilization of myeloid-derived suppressor cells. Cancer Res..

[84] Chiarugi A, Dolle C, Felici R, Ziegler M (2012). The NAD metabolome-a key determinant of cancer cell biology. Nat. Rev. Cancer.

[85] Ghosh D, Levault KR, Brewer GJ (2014). Relative importance of redox buffers GSH and NAD(P)H in age-related neurodegeneration and Alzheimer disease-like mouse neurons. Aging Cell.

[86] Singh T, Newman AB (2011). Inflammatory markers in population studies of aging. Ageing Res. Rev..

[87] Burgos ES, Vetticatt MJ, Schramm VL (2013). Recycling nicotinamide. The transition-state structure of human nicotinamide phosphoribosyltransferase. J. Am. Chem. Soc..

[88] Lim JH (2010). Sirtuin 1 modulates cellular responses to hypoxia by deacetylating hypoxia-inducible factor 1alpha. Mol. Cell.

[89] Yang H (2012). SIRT1 activators suppress inflammatory responses through promotion of p65 deacetylation and inhibition of NF-kappaB activity. PLoS One.

[90] Audrito V (2015). Extracellular nicotinamide phosphoribosyltransferase (NAMPT) promotes M2 macrophage polarization in chronic lymphocytic leukemia. Blood.

[91] Liu G (2014). SIRT1 limits the function and fate of myeloid-derived suppressor cells in tumors by orchestrating HIF-1alpha-dependent glycolysis. Cancer Res..

[92] Skokowa J (2009). NAMPT is essential for the G-CSF-induced myeloid differentiation via a NAD(+)-sirtuin-1-dependent pathway. Nat. Med..

[93] Young MR, Ihm J, Lozano Y, Wright MA, Prechel MM (1995). Treating tumor-bearing mice with vitamin D3 diminishes tumor-induced myelopoiesis and associated immunosuppression, and reduces tumor metastasis and recurrence. Cancer Immunol. Immunother..

[94] Lathers DM, Clark JI, Achille NJ, Young MR (2004). Phase 1B study to improve immune responses in head and neck cancer patients using escalating doses of 25-hydroxyvitamin D3. Cancer Immunol. Immunother..

[95] Chang YH, Chang DM, Lin KC, Shin SJ, Lee YJ (2011). Visfatin in overweight/obesity, type 2 diabetes mellitus, insulin resistance, metabolic syndrome and cardiovascular diseases: a meta-analysis and systemic review. Diabetes Metab. Res. Rev..

[96] Sica A, Mantovani A (2012). Macrophage plasticity and polarization: in vivo veritas. J. Clin. Investig..

[97] Haschemi A (2012). The sedoheptulose kinase CARKL directs macrophage polarization through control of glucose metabolism. Cell Metab..

[98] Jha AK (2015). Network integration of parallel metabolic and transcriptional data reveals metabolic modules that regulate macrophage polarization. Immunity.

[99] Blagih J, Jones RG (2012). Polarizing macrophages through reprogramming of glucose metabolism. Cell Metab..

[100] Vats D (2006). Oxidative metabolism and PGC-1beta attenuate macrophage-mediated inflammation. Cell Metab..

[101] Odegaard JI (2008). Alternative M2 activation of Kupffer cells by PPARdelta ameliorates obesity-induced insulin resistance. Cell Metab..

[102] Kang K (2008). Adipocyte-derived Th2 cytokines and myeloid PPARdelta regulate macrophage polarization and insulin sensitivity. Cell Metab..

[103] Pearce EJ, Everts B (2015). Dendritic cell metabolism. Nat. Rev. Immunol..

[104] Yang L, Carbone DP (2004). Tumor-host immune interactions and dendritic cell dysfunction. Adv. Cancer Res..

[105] Oguro H (2017). 27-Hydroxycholesterol induces hematopoietic stem cell mobilization and extramedullary hematopoiesis during pregnancy. J. Clin. Investig..

[106] Traversari C, Sozzani S, Steffensen KR, Russo V (2014). LXR-dependent and -independent effects of oxysterols on immunity and tumor growth. Eur. J. Immunol..

[107] Bensinger SJ, Tontonoz P (2008). Integration of metabolism and inflammation by lipid-activated nuclear receptors. Nature.

[108] Villablanca EJ (2010). Tumor-mediated liver X receptor-alpha activation inhibits CC chemokine receptor-7 expression on dendritic cells and dampens antitumor responses. Nat. Med.

[109] Jiang L, Fang X, Wang H, Li D, Wang X (2018). Ovarian cancer-intrinsic fatty acid synthase prevents anti-tumor immunity by disrupting tumor-infiltrating dendritic cells. Front Immunol..

[110] Nguyen NT (2010). Aryl hydrocarbon receptor negatively regulates dendritic cell immunogenicity via a kynurenine-dependent mechanism. Proc. Natl Acad. Sci. USA.

[111] Krawczyk CM (2010). Toll-like receptor-induced changes in glycolytic metabolism regulate dendritic cell activation. Blood.

[112] Everts B (2014). TLR-driven early glycolytic reprogramming via the kinases TBK1-IKKvarepsilon supports the anabolic demands of dendritic cell activation. Nat. Immunol..

[113] Xie H, Simon MC (2017). Oxygen availability and metabolic reprogramming in cancer. J. Biol. Chem..

[114] Zhang Z (2010). Antigen presentation by dendritic cells in tumors is disrupted by altered metabolism that involves pyruvate kinase M2 and its interaction with SOCS3. Cancer Res.

[115] Gottfried E (2006). Tumor-derived lactic acid modulates dendritic cell activation and antigen expression. Blood.

[116] Colegio OR (2014). Functional polarization of tumour-associated macrophages by tumour-derived lactic acid. Nature.

[117] Ghiringhelli F (2009). Activation of the NLRP3 inflammasome in dendritic cells induces IL-1beta-dependent adaptive immunity against tumors. Nat. Med.

[118] Kayhan M, Koyas A, Akdemir I, Savas AC, Cekic C (2019). Adenosine receptor signaling targets both PKA and Epac pathways to polarize dendritic cells to a suppressive phenotype. J. Immunol..

[119] Zitvogel L (2010). Immunogenic tumor cell death for optimal anticancer therapy: the calreticulin exposure pathway. Clin. Cancer Res..

[120] Coffelt SB, Wellenstein MD, de Visser KE (2016). Neutrophils in cancer: neutral no more. Nat. Rev. Cancer.

[121] Colotta F, Re F, Polentarutti N, Sozzani S, Mantovani A (1992). Modulation of granulocyte survival and programmed cell death by cytokines and bacterial products. Blood.

[122] van Raam BJ, Drewniak A, Groenewold V, van den Berg TK, Kuijpers TW (2008). Granulocyte colony-stimulating factor delays neutrophil apoptosis by inhibition of calpains upstream of caspase-3. Blood.

[123] Gerrard TL, Cohen DJ, Kaplan AM (1981). Human neutrophil-mediated cytotoxicity to tumor cells. J. Natl Cancer Inst..

[124] Coffelt SB (2015). IL-17-producing gammadelta T cells and neutrophils conspire to promote breast cancer metastasis. Nature.

[125] He G (2015). Peritumoural neutrophils negatively regulate adaptive immunity via the PD-L1/PD-1 signalling pathway in hepatocellular carcinoma. J. Exp. Clin. Cancer Res..

[126] Peng B, Wang YH, Liu YM, Ma LX (2015). Prognostic significance of the neutrophil to lymphocyte ratio in patients with non-small cell lung cancer: a systemic review and meta-analysis. Int J. Clin. Exp. Med..

[127] Gentles AJ (2015). The prognostic landscape of genes and infiltrating immune cells across human cancers. Nat. Med..

[128] Fridlender ZG (2009). Polarization of tumor-associated neutrophil phenotype by TGF-beta: "N1" versus "N2" TAN. Cancer Cell.

[129] Hudome S (1997). The role of neutrophils in the production of hypoxic-ischemic brain injury in the neonatal rat. Pediatr. Res..

[130] Dieterich HJ, Weissmuller T, Rosenberger P, Eltzschig HK (2006). Effect of hydroxyethyl starch on vascular leak syndrome and neutrophil accumulation during hypoxia. Crit. Care Med..

[131] Melillo G (1995). A hypoxia-responsive element mediates a novel pathway of activation of the inducible nitric oxide synthase promoter. J. Exp. Med..

[132] Munn DH (2005). GCN2 kinase in T cells mediates proliferative arrest and anergy induction in response to indoleamine 2,3-dioxygenase. Immunity.

[133] Fletcher M (2015). l-Arginine depletion blunts antitumor T-cell responses by inducing myeloid-derived suppressor cells. Cancer Res..

[134] Sharda DR (2011). Regulation of macrophage arginase expression and tumor growth by the Ron receptor tyrosine kinase. J. Immunol..

[135] Kusmartsev S, Gabrilovich DI (2006). Effect of tumor-derived cytokines and growth factors on differentiation and immune suppressive features of myeloid cells in cancer. Cancer Metastasis Rev..

[136] Sica A, Bronte V (2007). Altered macrophage differentiation and immune dysfunction in tumor development. J. Clin. Investig..

[137] Srivastava MK, Sinha P, Clements VK, Rodriguez P, Ostrand-Rosenberg S (2010). Myeloid-derived suppressor cells inhibit T-cell activation by depleting cystine and cysteine. Cancer Res..

[138] Hotamisligil GS, Shargill NS, Spiegelman BM (1993). Adipose expression of tumor necrosis factor-alpha: direct role in obesity-linked insulin resistance. Science.

[139] Deng T, Lyon CJ, Bergin S, Caligiuri MA, Hsueh WA (2016). Obesity, inflammation, and cancer. Annu Rev. Pathol..

[140] Cowey S, Hardy RW (2006). The metabolic syndrome: a high-risk state for cancer?. Am. J. Pathol..

[141] Sandhu MS, Dunger DB, Giovannucci EL (2002). Insulin, insulin-like growth factor-I (IGF-I), IGF binding proteins, their biologic interactions, and colorectal cancer. J. Natl Cancer Inst..

[142] Aguirre GA, De Ita JR, de la Garza RG, Castilla-Cortazar I (2016). Insulin-like growth factor-1 deficiency and metabolic syndrome. J. Transl. Med..

[143] Braga TT, Agudelo JS, Camara NO (2015). Macrophages during the fibrotic process: M2 as friend and foe. Front Immunol..

[144] Weisberg SP (2003). Obesity is associated with macrophage accumulation in adipose tissue. J. Clin. Investig..

[145] Hillers-Ziemer LE (2020). Obesity promotes cooperation of cancer stem-like cells and macrophages to enhance mammary tumor angiogenesis. Cancers.

[146] Balkwill F, Mantovani A (2001). Inflammation and cancer: back to Virchow?. Lancet.

[147] Al Sayed MF (2019). T-cell-secreted TNFalpha induces emergency myelopoiesis and myeloid-derived suppressor cell differentiation in cancer. Cancer Res.

[148] Goodwin PJ (2002). Fasting insulin and outcome in early-stage breast cancer: results of a prospective cohort study. J. Clin. Oncol..

[149] Borugian MJ (2004). Insulin, macronutrient intake, and physical activity: are potential indicators of insulin resistance associated with mortality from breast cancer?. Cancer Epidemiol. Biomark. Prev..

[150] Shoelson SE, Lee J, Goldfine AB (2006). Inflammation and insulin resistance. J. Clin. Investig..

[151] Goodwin PJ (2009). High insulin levels in newly diagnosed breast cancer patients reflect underlying insulin resistance and are associated with components of the insulin resistance syndrome. Breast Cancer Res. Treat..

[152] Gennari A (2020). Insulin-like growth factor-1 receptor (IGF-1R) expression on circulating tumor cells (CTCs) and metastatic breast cancer outcome: results from the TransMYME trial. Breast Cancer Res. Treat..

[153] Zakikhani M, Dowling R, Fantus IG, Sonenberg N, Pollak M (2006). Metformin is an AMP kinase-dependent growth inhibitor for breast cancer cells. Cancer Res..

[154] Goodwin PJ (2015). Effect of Metformin vs Placebo on Weight and Metabolic Factors in NCIC CTG MA.3.2.. J. Natl Cancer Inst..

[155] Nanni O (2019). Metformin plus chemotherapy versus chemotherapy alone in the first-line treatment of HER2-negative metastatic breast cancer. The MYME randomized, phase 2 clinical trial. Breast Cancer Res. Treat..

[156] Kotwal A, Haddox C, Block M, Kudva YC (2019). Immune checkpoint inhibitors: an emerging cause of insulin-dependent diabetes. BMJ Open Diabetes Res. Care.

[157] McQuade JL (2018). Association of body-mass index and outcomes in patients with metastatic melanoma treated with targeted therapy, immunotherapy, or chemotherapy: a retrospective, multicohort analysis. Lancet Oncol..

[158] Donnelly D (2019). The complex relationship between body mass index and response to immune checkpoint inhibition in metastatic melanoma patients. J. Immunother. Cancer.

[159] Cheng SC (2014). mTOR- and HIF-1alpha-mediated aerobic glycolysis as metabolic basis for trained immunity. Science.

[160] O’Neill LA, Hardie DG (2013). Metabolism of inflammation limited by AMPK and pseudo-starvation. Nature.

[161] Husain Z, Seth P, Sukhatme VP (2013). Tumor-derived lactate and myeloid-derived suppressor cells: linking metabolism to cancer immunology. Oncoimmunology.

[162] Husain Z, Huang Y, Seth P, Sukhatme VP (2013). Tumor-derived lactate modifies antitumor immune response: effect on myeloid-derived suppressor cells and NK cells. J. Immunol..

[163] Latham T (2012). Lactate, a product of glycolytic metabolism, inhibits histone deacetylase activity and promotes changes in gene expression. Nucleic Acids Res..

[164] Li W (2018). Aerobic glycolysis controls myeloid-derived suppressor cells and tumor immunity via a specific CEBPB isoform in triple-negative breast cancer. Cell Metab..

[165] Salminen A, Kauppinen A, Kaarniranta K (2019). AMPK activation inhibits the functions of myeloid-derived suppressor cells (MDSC): impact on cancer and aging. J. Mol. Med..

[166] Baumann T (2020). Regulatory myeloid cells paralyze T cells through cell-cell transfer of the metabolite methylglyoxal. Nat. Immunol..

[167] Allaman I, Belanger M, Magistretti PJ (2015). Methylglyoxal, the dark side of glycolysis. Front Neurosci..

[168] Unoki H, Yamagishi S (2008). Advanced glycation end products and insulin resistance. Curr. Pharm. Des..

[169] Chang X, Wei C (2011). Glycolysis and rheumatoid arthritis. Int J. Rheum. Dis..

[170] Stathopoulou C, Nikoleri D, Bertsias G (2019). Immunometabolism: an overview and therapeutic prospects in autoimmune diseases. Immunotherapy.

[171] Lenihan DJ, Cardinale DM (2012). Late cardiac effects of cancer treatment. J. Clin. Oncol..

[172] de Haas EC (2010). The metabolic syndrome in cancer survivors. Lancet Oncol..

[173] Van Gaal LF, Mertens IL, De Block CE (2006). Mechanisms linking obesity with cardiovascular disease. Nature.

[174] Kroenke CH, Chen WY, Rosner B, Holmes MD (2005). Weight, weight gain, and survival after breast cancer diagnosis. J. Clin. Oncol..

[175] Rosen GP, Nguyen HT, Shaibi GQ (2013). Metabolic syndrome in pediatric cancer survivors: a mechanistic review. Pediatr. Blood Cancer.

[176] Floyd J, Mirza I, Sachs B, Perry MC (2006). Hepatotoxicity of chemotherapy. Semin. Oncol..

[177] Dieli-Conwright CM (2016). An observational study to examine changes in metabolic syndrome components in patients with breast cancer receiving neoadjuvant or adjuvant chemotherapy. Cancer.

[178] Morel S (2017). Lipid and lipoprotein abnormalities in acute lymphoblastic leukemia survivors. J. Lipid Res..

[179] van Waas M (2012). Abdominal radiotherapy: a major determinant of metabolic syndrome in nephroblastoma and neuroblastoma survivors. PLoS One.

[180] Shimizu Y, Kato H, Schull WJ (1990). Studies of the mortality of A-bomb survivors. 9. Mortality, 1950-1985: Part 2. Cancer mortality based on the recently revised doses (DS86). Radiat. Res.

[181] de Vathaire F (2012). Radiation dose to the pancreas and risk of diabetes mellitus in childhood cancer survivors: a retrospective cohort study. Lancet Oncol..

[182] Janiszewski PM (2007). Abdominal obesity, liver fat, and muscle composition in survivors of childhood acute lymphoblastic leukemia. J. Clin. Endocrinol. Metab..

[183] Ding ZC, Munn DH, Zhou G (2014). Chemotherapy-induced myeloid suppressor cells and antitumor immunity: the Janus face of chemotherapy in immunomodulation. Oncoimmunology.

[184] Ding ZC (2014). Immunosuppressive myeloid cells induced by chemotherapy attenuate antitumor CD4+ T-cell responses through the PD-1-PD-L1 axis. Cancer Res..

[185] Kodumudi KN (2010). A novel chemoimmunomodulating property of docetaxel: suppression of myeloid-derived suppressor cells in tumor bearers. Clin. Cancer Res..

[186] Vincent J (2010). 5-Fluorouracil selectively kills tumor-associated myeloid-derived suppressor cells resulting in enhanced T cell-dependent antitumor immunity. Cancer Res..

[187] Suzuki E, Kapoor V, Jassar AS, Kaiser LR, Albelda SM (2005). Gemcitabine selectively eliminates splenic Gr-1+/CD11b+ myeloid suppressor cells in tumor-bearing animals and enhances antitumor immune activity. Clin. Cancer Res..

[188] Sistigu A (2011). Immunomodulatory effects of cyclophosphamide and implementations for vaccine design. Semin. Immunopathol..

[189] Pelaez B, Campillo JA, Lopez-Asenjo JA, Subiza JL (2001). Cyclophosphamide induces the development of early myeloid cells suppressing tumor cell growth by a nitric oxide-dependent mechanism. J. Immunol..

[190] Mikyskova R (2012). Cyclophosphamide-induced myeloid-derived suppressor cell population is immunosuppressive but not identical to myeloid-derived suppressor cells induced by growing TC-1 tumors. J. Immunother..

[191] Bass KK, Mastrangelo MJ (1998). Immunopotentiation with low-dose cyclophosphamide in the active specific immunotherapy of cancer. Cancer Immunol. Immunother..

[192] Wada S (2009). Cyclophosphamide augments antitumor immunity: studies in an autochthonous prostate cancer model. Cancer Res..

[193] Diaz-Montero CM (2009). Increased circulating myeloid-derived suppressor cells correlate with clinical cancer stage, metastatic tumor burden, and doxorubicin-cyclophosphamide chemotherapy. Cancer Immunol. Immunother..

[194] Wesolowski R (2017). Circulating myeloid-derived suppressor cells increase in patients undergoing neo-adjuvant chemotherapy for breast cancer. Cancer Immunol. Immunother..

[195] Kaewkangsadan V (2016). Crucial contributions by T lymphocytes (effector, regulatory, and checkpoint inhibitor) and cytokines (TH1, TH2, and TH17) to a pathological complete response induced by neoadjuvant chemotherapy in women with breast cancer. J. Immunol. Res.

[196] Gabitass RF, Annels NE, Stocken DD, Pandha HA, Middleton GW (2011). Elevated myeloid-derived suppressor cells in pancreatic, esophageal and gastric cancer are an independent prognostic factor and are associated with significant elevation of the Th2 cytokine interleukin-13. Cancer Immunol. Immunother..

[197] Isherwood J (2020). Myeloid derived suppressor cells are reduced and T regulatory cells stabilised in patients with advanced pancreatic cancer treated with gemcitabine and intravenous omega 3. Ann. Transl. Med..

[198] Fallah, J. et al. Myeloid-derived suppressor cells in nonmetastatic urothelial carcinoma of bladder is associated with pathologic complete response and overall survival. *Clin. Genitourin. Cancer*10.1016/j.clgc.2020.03.004 (2020). 10.1016/j.clgc.2020.03.004 32340875

[199] Koinis F (2016). Effect of first-line treatment on myeloid-derived suppressor cells’ subpopulations in the peripheral blood of patients with non-small cell lung cancer. J. Thorac. Oncol..

[200] Gandhi L (2018). Pembrolizumab plus chemotherapy in metastatic non-small-cell lung cancer. N. Engl. J. Med..

[201] Schmid P (2018). Atezolizumab and nab-paclitaxel in advanced triple-negative breast cancer. N. Engl. J. Med..

[202] Heinhuis KM (2019). Enhancing antitumor response by combining immune checkpoint inhibitors with chemotherapy in solid tumors. Ann. Oncol..

[203] Brahmer J (2015). Nivolumab versus docetaxel in advanced squamous-cell non-small-cell lung cancer. N. Engl. J. Med..

[204] Ferrara R (2018). Hyperprogressive disease in patients with advanced non-small cell lung cancer treated with PD-1/PD-L1 inhibitors or with single-agent chemotherapy. JAMA Oncol..

[205] Zhang S (2016). The role of myeloid-derived suppressor cells in patients with solid tumors: a meta-analysis. PLoS One.

[206] Gonda K (2017). Myeloid-derived suppressor cells are increased and correlated with type 2 immune responses, malnutrition, inflammation, and poor prognosis in patients with breast cancer. Oncol. Lett..

[207] Martens A (2016). Baseline peripheral blood biomarkers associated with clinical outcome of advanced melanoma patients treated with ipilimumab. Clin. Cancer Res..

[208] Bronte V (2016). Recommendations for myeloid-derived suppressor cell nomenclature and characterization standards. Nat. Commun..

[209] Alkasalias T, Moyano-Galceran L, Arsenian-Henriksson M, Lehti K (2018). Fibroblasts in the tumor microenvironment: shield or spear?. Int J. Mol. Sci..

[210] Talmadge JE, Gabrilovich DI (2013). History of myeloid-derived suppressor cells. Nat. Rev. Cancer.

[211] Meyer C (2014). Frequencies of circulating MDSC correlate with clinical outcome of melanoma patients treated with ipilimumab. Cancer Immunol. Immunother..

[212] Santegoets SJ (2014). Myeloid derived suppressor and dendritic cell subsets are related to clinical outcome in prostate cancer patients treated with prostate GVAX and ipilimumab. J. Immunother. Cancer.

[213] Nefedova Y (2007). Mechanism of all-trans retinoic acid effect on tumor-associated myeloid-derived suppressor cells. Cancer Res..

[214] Kusmartsev S (2008). Reversal of myeloid cell-mediated immunosuppression in patients with metastatic renal cell carcinoma. Clin. Cancer Res..

[215] Won WJ, Deshane JS, Leavenworth JW, Oliva CR, Griguer CE (2019). Metabolic and functional reprogramming of myeloid-derived suppressor cells and their therapeutic control in glioblastoma. Cell Stress.

[216] Lee JM (2012). The restoration of myeloid-derived suppressor cells as functional antigen-presenting cells by NKT cell help and all-trans-retinoic acid treatment. Int J. Cancer.

[217] Sarrazy V (2016). Disruption of Glut1 in hematopoietic stem cells prevents myelopoiesis and enhanced glucose flux in atheromatous plaques of ApoE(-/-) mice. Circ. Res..

[218] Strauss L (2020). Targeted deletion of PD-1 in myeloid cells induces antitumor immunity. Sci. Immunol..

[219] Saraei P, Asadi I, Kakar MA, Moradi-Kor N (2019). The beneficial effects of metformin on cancer prevention and therapy: a comprehensive review of recent advances. Cancer Manag. Res..

[220] Qin G (2018). Metformin blocks myeloid-derived suppressor cell accumulation through AMPK-DACH1-CXCL1 axis. Oncoimmunology.

[221] Xu P (2019). Metformin inhibits the function of granulocytic myeloid-derived suppressor cells in tumor-bearing mice. Biomed. Pharmacother..

[222] Leone RD (2019). Glutamine blockade induces divergent metabolic programs to overcome tumor immune evasion. Science.

